# Supplementary figures and images for: Network analysis of patient flow in two UK acute care hospitals identifies key sub-networks for A&E performance
Source: PLoS One. 2017 Oct 2;12(10):e0185912. doi: 10.1371/journal.pone.0185912 (PMC5624623; doi:10.1371/journal.pone.0185912)

**a DH**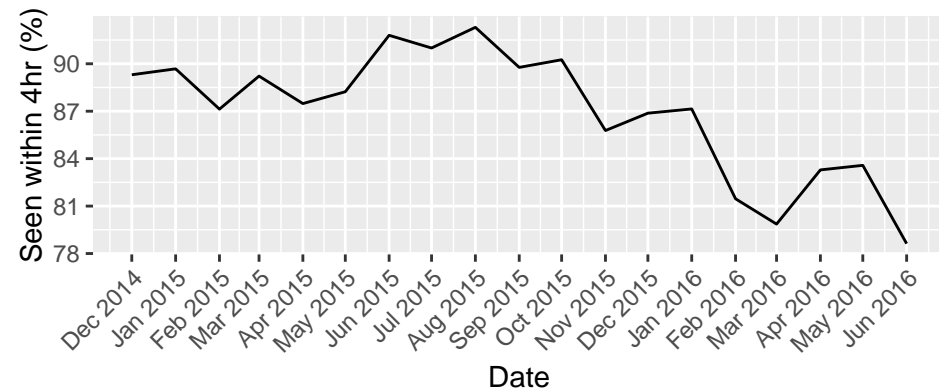**b PRUH**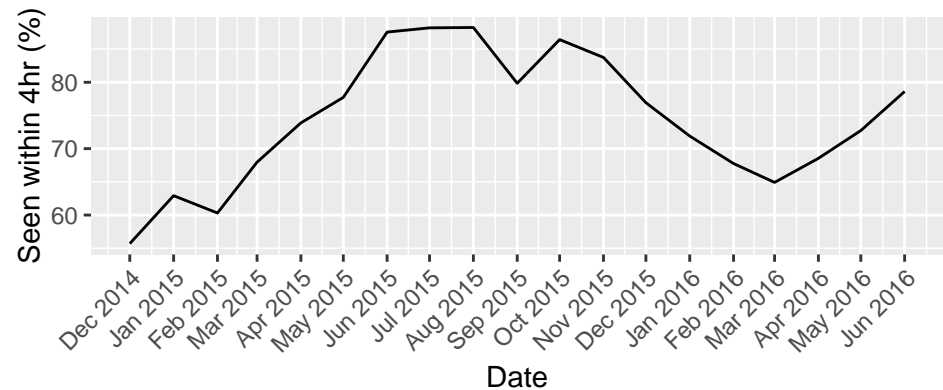**c DH  $r^2 = 0.655$** 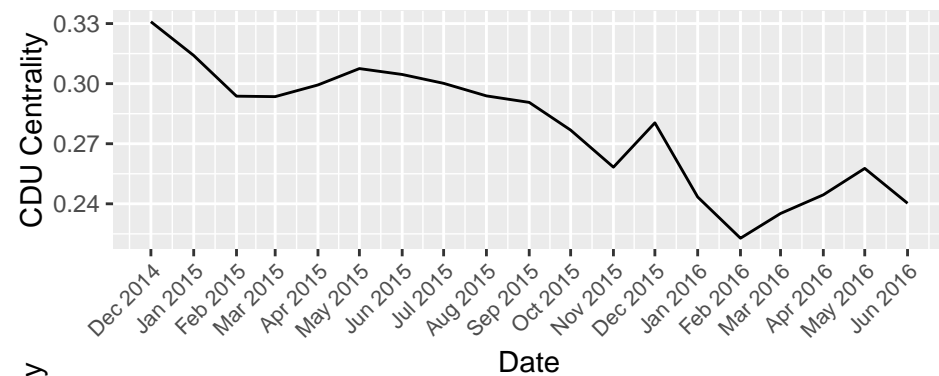**d PRUH  $r^2 = 0.463$** 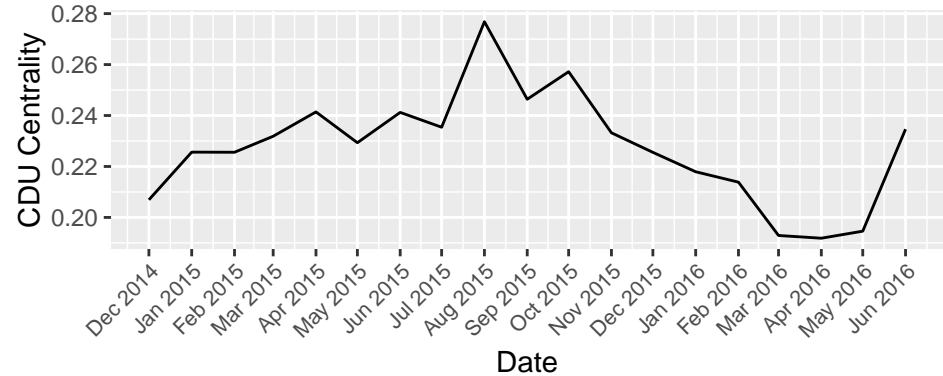**e DH  $r^2 = 0.68$** 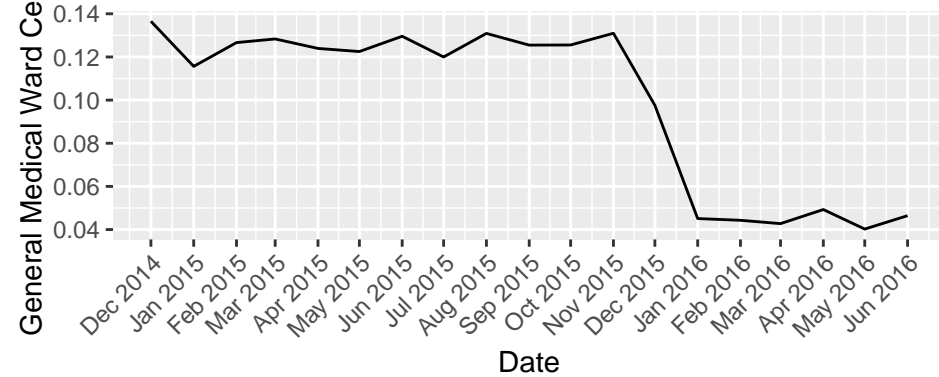**f PRUH  $r^2 = 0.575$** 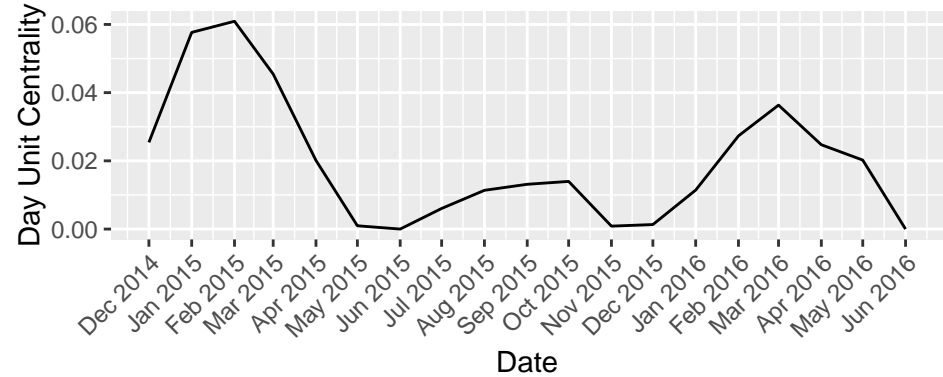

Supplement: S1 Fig — (a, b) Mean monthly A&E performance over time for each site, as measured by the percent of patient meeting the 4 hour A&E waiting time target. (c-f) Centrality of wards over time. Here centrality is defined as the proportion of all observed paths each node lies on per month. This measure was chosen as shortest paths do not reflect actual patient trajectories. (e) this general medical ward was re-organised during Dec 2015 to try to cope with winter pressures. R-squared values are shown for the correlation between the monthly centrality of each ward and the monthly performance of the corresponding hospital A&E department. DH = Denmark Hill, PRUH = Princess Royal University Hospital, CDU = Clinical Decision Unit. (PDF) [file pone.0185912.s001.pdf]

**a** *PRUH*

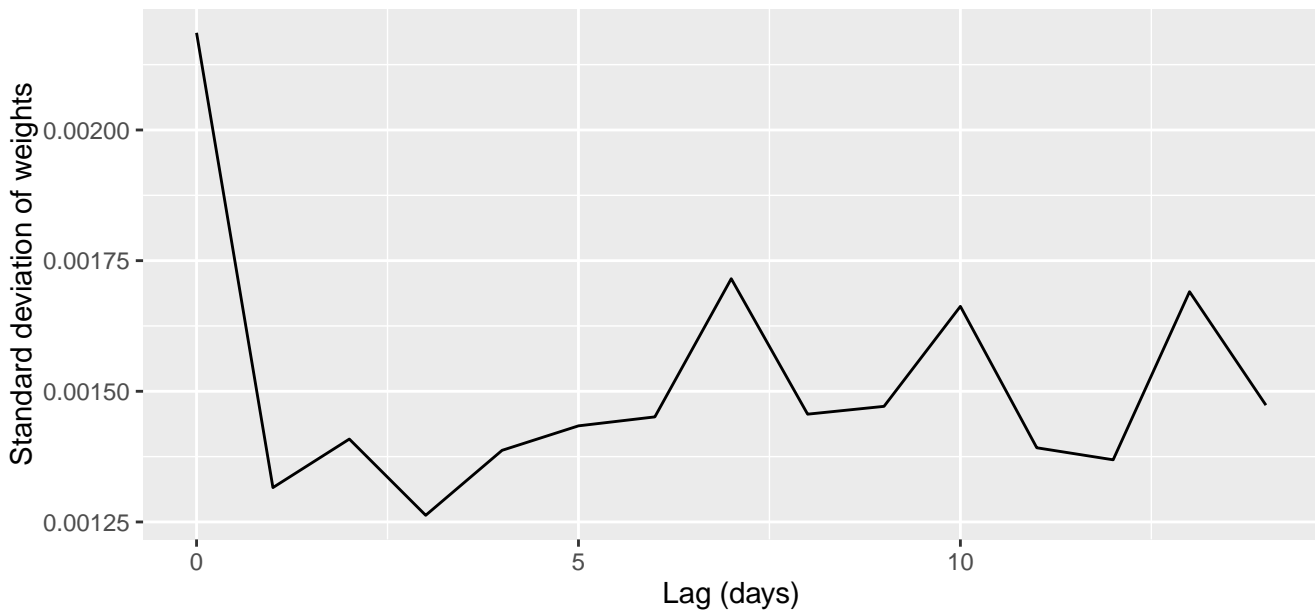

**b** *DH*

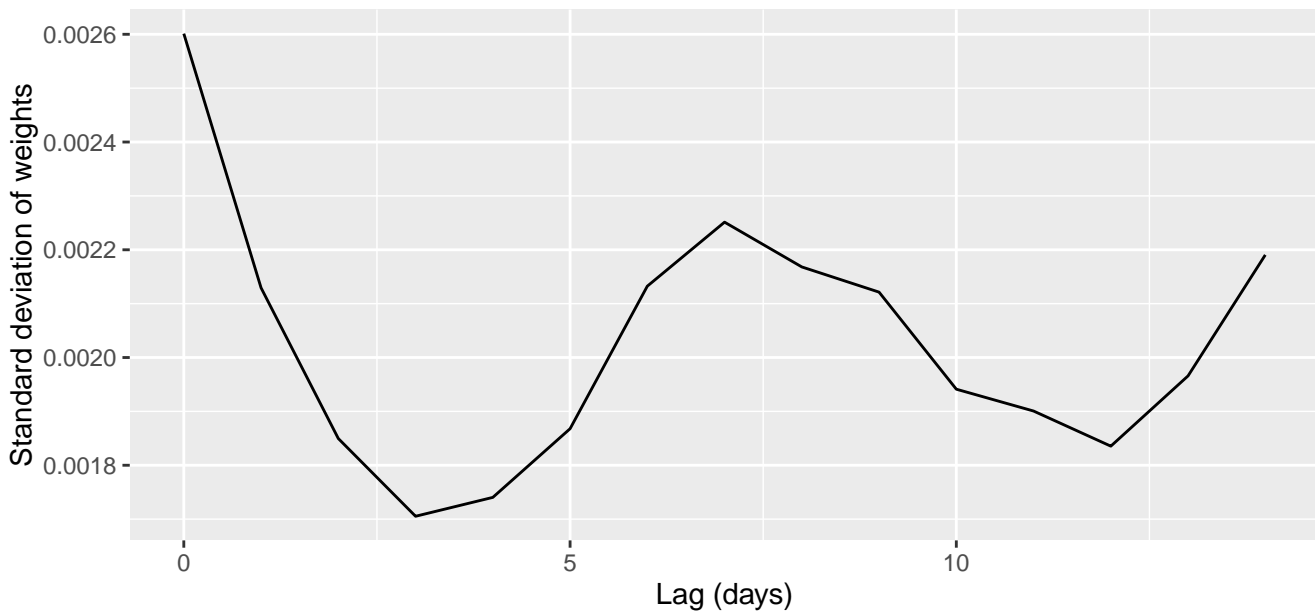

Supplement: S2 Fig — (a, b) A series of differential patient flow networks (comparing the High (top 10%) and Low (bottom 10%) performance groups) was created for each site by applying an increasingly large lag to the flow data e.g. using the patient flow observed 3 days prior to the days in each performance group. A lag of 0 indicates the same day, a lag of 7 is one week earlier. Networks were generated for all lag values from 0 to 14 days and the standard deviation over all edges in each network is shown. PRUH = Princess Royal University Hospital, DH = Denmark Hill. (PDF) [file pone.0185912.s002.pdf]
